# Supplementary material for: Prostate cancer in Spain: A retrospective database analysis of hospital incidence and the direct medical costs
Source: PLoS One. 2024 Mar 7;19(3):e0298764. doi: 10.1371/journal.pone.0298764 (PMC10919697; doi:10.1371/journal.pone.0298764)
Supplement: Attachment — Submitted filename: Response to reviewers.docx [file pone.0298764.s001.docx]

Reviewers' comments:

Reviewer's Responses to Questions

**Comments to the Author**

1. Is the manuscript technically sound, and do the data support the conclusions?

The manuscript must describe a technically sound piece of scientific research with data that supports the conclusions. Experiments must have been conducted rigorously, with appropriate controls, replication, and sample sizes. The conclusions must be drawn appropriately based on the data presented.

Reviewer #1: No

Reviewer #2: Yes

2. Has the statistical analysis been performed appropriately and rigorously?

Reviewer #1: No

Reviewer #2: Yes

3. Have the authors made all data underlying the findings in their manuscript fully available?

The [PLOS Data policy](http://www.plosone.org/static/policies.action#sharing) requires authors to make all data underlying the findings described in their manuscript fully available without restriction, with rare exception (please refer to the Data Availability Statement in the manuscript PDF file). The data should be provided as part of the manuscript or its supporting information, or deposited to a public repository. For example, in addition to summary statistics, the data points behind means, medians and variance measures should be available. If there are restrictions on publicly sharing data—e.g. participant privacy or use of data from a third party—those must be specified.

Reviewer #1: No

Reviewer #2: Yes

4. Is the manuscript presented in an intelligible fashion and written in standard English?

PLOS ONE does not copyedit accepted manuscripts, so the language in submitted articles must be clear, correct, and unambiguous. Any typographical or grammatical errors should be corrected at revision, so please note any specific errors here.

Reviewer #1: Yes

Reviewer #2: Yes

5. Review Comments to the Author

Please use the space provided to explain your answers to the questions above. You may also include additional comments for the author, including concerns about dual publication, research ethics, or publication ethics. (Please upload your review as an attachment if it exceeds 20,000 characters)

Reviewer #1: The authors have conducted a good study, however, there are a lot of important and relevant information missing from the manuscript.

Firstly, the authors describe the study as a multi center study but have not mentioned how many centers the data were collected from to justify their sample size.

*The data come from a database that includes information from many centers, but we cannot provide the exact number of centers from which our data come. As indicated in the text, the database contains information from 90% of the hospitals in Spain and includes data from all regions.*

Secondly, the authors have not mentioned in detail the type of diagnostic tests, if any, that the patients were subjected to and the costs of these tests and wether or not the patients paid for these themselves.

*The subjects were not included based on any diagnostic test. The patients included in the study came from a database and were selected according to its diagnosis based on IDC 10 codes.*

Thirdly, it will be beneficial for the authors to highlight the major treatment procedures that these patients underwent and the average cost of such procedures and wether or not the patients had multiple procedures done during the course of their treatments.

*The most common medical procedures have already been provided in the results section and in Table 3. Regarding costs, the database just provides the total cost of the admission, so the procedure cost is included in the total, but we can not know the exact cost of the concrete procedure. Additional information on the number of procedures performed per patient has been added.*

The authors should also mention wether the patients benefit from any health insurance schemes if available.

*Information about this point has been added. In Spain, healthcare is free of charge, so the costs provided will be those paid by the national health system.*

Overall, I believe this study can be made better and more impactful than it currently is.

Reviewer #2: The paper is solid and make a point asPSA merges as an important predictor according to this study. The results are solid however the authors should clarify which software they have used for each statistical analysis.

*Just Stata SE has been used to conduct the statistical analysis. This information has been updated in the methods section.*
